# A case of poetry therapy interventions for a patient with schizotypal personality disorder

**DOI:** 10.1192/j.eurpsy.2026.11917

**Published:** 2026-07-31

**Authors:** E. L. Nikolaev, F. V. Orlov, E. F. Orlova, H. Kumar

**Affiliations:** 1 Department of Social and Clinical Psychology; 2 Department of Psychiatry, Medical Psychology and Neurology; 3 Medical Faculty, I.N. Ulianov Chuvash State University, Cheboksary, Russian Federation

## Abstract

**Introduction:**

Schizotypal personality disorder represents a broad range of maladaptive behavior, which has been linked to both personality disorder and schizophrenia spectrum disorders (Cheli et al., 2019). Psychotherapy may support and assist patients with schizotypal personality disorder (Nielsen et al., 2023), however, to date, the practice of psychosocial approaches to its treatment is not sufficiently developed (Cheli et al., 2019). Meanwhile, poetry therapy is widely used for a wide range of emotional and mental disorders.

**Objectives:**

Our aim is to present a clinical case and review of published literature to reflect the importance of using poetry therapy interventions in order to alleviate the severity of negative symptoms of mental disorder.

**Methods:**

Clinical case report and literature review based on PubMed search.

**Results:**

A 34-year old patient with schizotypal personality disorder recalls his complains of weakness, lassitude, loss of strength, lack of interest, and inactivity during his first visit to a psychiatrist at the age of 18. Further, he feels decline in activity and lassitude. The clinical picture shows prevailing depressive symptoms with a constant feeling of tension and constraint, and episodes of anxiety and apathy. For the last 8 years, he has been undergoing an out-patient treatment. Additionally to pharmaceutic therapy, he is having an individual and group psychotherapy, social skills training, and poetry therapy. The complex psychosocial intervention has the aim to booster activity, enhance social interaction, broaden the behavioral repertoire, and increase achievement motivation. The specialists encourage the patient to lean on resources that require creativity by turning to poetry. Writing poetry promotes directed activation of the processes of inner development and solution of intrapersonal problems. In the course of therapy, we also set the goal of forming constructive guidelines aimed at overcoming the person’s belief in impossibility of achieving success. Poetry therapy interventions helped to lead the patient into the context of searching the meaning in life inside and outside himself and changing his attitude to himself and the world around him. We noticed positive dynamics of his emotional state, cognitive guidelines, and behavioral activity. The goal of the therapy has changed from accentuating on negative symptoms to boosting his social interaction. The patient has learned to deal with his fears, he feels ready to speak before the audience, write poems for contests and publications.

**Conclusions:**

The given clinical case demonstrates that use of poetry therapy interventions in treating patients with schizotypal personality disorder can be an effective means of alleviating the negative symptoms and enhancing the level of their social adaptation.

**Disclosure of Interest:**

None Declared

